# Association between self-efficacy, social support, knowledge of COVID-19, COVID-19 perception and stress, anxiety, depression of healthcare providers: the role of news media exposure as moderator

**DOI:** 10.1192/j.eurpsy.2022.1257

**Published:** 2022-09-01

**Authors:** A. Prouzou, E. Aslani, V. Spinaris, G. Lyrakos

**Affiliations:** 1 City Unity College, Psychology, Athens, Greece; 2 General Hospital Nikaia Ag. Panteleimon, 2nd Covid Clinic, Nikaia, Greece; 3 General Hospital of Nikaia, Psychiatric, Nikaia, Greece

**Keywords:** Covid-19, media exposure, Stress, health care providers

## Abstract

**Introduction:**

All pandemic outbreaks because of their rapid spread and high mortality rate cause to everyone considerable stress and anxiety.

**Objectives:**

The aim of the present study is to investigate how news media exposure moderates the relationship between stress, anxiety, depression and self-efficacy, social support, knowledge of the coronavirus and coronavirus perception.

**Methods:**

223 healthcare providers, men 46 (20.6%) and women 177 (79.4%), working in hospitals in Greece participated in the study. independent t-test, one-way ANOVAs, Pearson’s correlation, multiple-linear regression and moderator’s analysis were analyzed with SPSS23.

**Results:**

Organization support, friends support, covid-19 knowledge and covid-19 perception are most significant predictors to stress, F (4,218) = 11.47, p < .001 and Adjusted R2- .159. Friends support, covid-19 knowledge and self-efficacy, working with covid19 patients and gender are most significant predictors to anxiety, F (5,217) = 11.16, p < .001 Adjusted R2- .186. Friends support, covid-19 knowledge and self-efficacy and organization support are most significant predictors to depression, F (4,218) = 16.37, p < .001 Adjusted R2-squared: .217. News media exposure did moderate the predictive power of almost all predictors for stress, anxiety and depression, at p<.05.

**Conclusions:**

Therefore, the study verifies previous findings arguing that stress, anxiety and depression are strongly associated with numerous factors. These associations seem to be moderated by news media exposure. It is recommended to further explore the impact news media exposure has during crucial periods, such as covid-19 outbreak.

**Disclosure:**

No significant relationships.

